# Different tibial rotational axes can be applied in combination according to the tibial tuberosity–posterior cruciate ligament distance in total knee arthroplasty

**DOI:** 10.1186/s12891-022-05859-9

**Published:** 2022-10-10

**Authors:** Le-Shu Zhang, Hang Zhou, Jin-Cheng Zhang, Qiang Zhang, Xiang-Yang Chen, Shuo Feng

**Affiliations:** Department of Orthopedic Surgery, Affiliated Hospital of Xuzhou Medical University, 99 Huaihai Road, Xuzhou, 221002 Jiangsu China

**Keywords:** Total knee arthroplasty, Rotational alignment, Tibial tuberosity, Tibial tuberosity–posterior cruciate ligament distance

## Abstract

**Purpose:**

The purpose of this study was to investigate whether tibial tuberosity–posterior cruciate ligament (TT-PCL) distance is representative of the true lateralization of tibial tuberosity in isolation and its influence on the accuracy of the Akagi line and medial third of the tibial tuberosity (MTTT).

**Methods:**

A total of 135 osteoarthritis patients with varus knees who undergoing computed tomography scans were enrolled to establish three-dimension models of the knees. Tibial width (TW), tibial tuberosity lateralization (TTL), posterior cruciate ligament lateralization (PCLL), knee rotation angle (KRA) and tibial rotational axes were measured and investigated their correlations with TT-PCL distance. Based on the analysis of receiver operating characteristic (ROC) curve, the influence of TT-PCL distance on the distributions of mismatch angles of tibial rotational axes was investigated with a safe zone (-5° to 10°).

**Results:**

TT-PCL distance was in significantly positive correlation with TW (*r* = 0.493; *P* < 0.001) and TTL (*r* = 0.378; *P* < 0.001) which was different with PCLL (*r* = 0.147; *P* = 0.009) and KRA (*r* = -0.166; *P* = 0.054). All tibial rotational axes were significantly positively correlated with TT-PCL distance (*P* < 0.001). The mismatch angles between the vertical line of the surgical epicondylar axis (SEA) and the Akagi line and MTTT were -1.7° ± 5.3° and 7.6° ± 5.6° respectively. In terms of the optimal cut-off value of 19 mm for TT-PCL distance, the Akagi line applied as tibial rotational axis ensures 87.3% of the positions of tibial components within the safe zone when TT-PCL distance > 19 mm, and MTTT ensures 83.3% when TT-PCL distance ≤ 19 mm.

**Conclusion:**

TT-PCL distances cannot reflect the true lateralization of tibial tuberosity in isolation but can aid in the combination of the Akagi line and MTTT in varus knees. The patients with TT-PCL distance > 19 mm are recommended to reference the Akagi line for tibial rotational alignment. MTTT is recommended to the patients with TT-PCL distance ≤ 19 mm. The study will aid surgeons in deciding which reference may be used by measuring TT-PCL distance using a preoperative CT.

## Introduction

Rotational alignment of tibial component is an important factor in determining the success of total knee arthroplasty. Rotational misalignment between femoral components and tibial components may cause various postoperative complications such as patellofemoral joint problems, gait abnormalities, polyethylene wear, stiff knees, or anterior knee pain [1–5]. Therefore, it is one of the most important goals to achieve correct rotational alignment of tibial components in total knee arthroplasty (TKA).

The consensus of femoral rotational alignment has been achieved by now where the surgical epicondylar axis (SEA), the posterior femoral condyle axis, and the Whiteside line have been shown to be reproducible and accurate as femoral rotational axes. Among them, the SEA which is the line connecting the prominent point of lateral femoral epicondyle and the medial femoral epicondylar sulcus moreover constitutes the flexion–extension axis of the knee [6]. As for tibial rotational alignment, although the researchers have proposed various reference axes for rotational alignment including medial border of the tibial tuberosity, medial third of the tibial tuberosity, the Akagi line, medial border of the patellar tendon at the attachment and medial sixth of the patellar tendon at the attachment [7–10]. However, the question of how to establish correct rotational alignment of tibial components remains unresolved. This is because the establishment of tibial rotational alignment axes is mostly based on the location of tibial tuberosity, whereas the lateralization of tibial tuberosity is highly variable among the OA knees [11, 12]. Howell et al. [12] found that the variation of the distance from medial third of the tibial tuberosity to medial border of the tibial tuberosity could be as high as 15 mm when using 80 mm as the standard tibial width by analyzing MRI scans of 115 knees. Eighty-six percent of the tibial components existed the mismatch angles exceeding 5° when reference MTTT.

The lateralization of the tibial tuberosity has been assessed by tibial tuberosity-trochlear groove (TT-TG) distance to adjust the choice of anatomical landmarks for tibial rotational alignment [13, 14]. However, TT-TG distance has its own limitations: First, it is suggested that TT-TG cannot be independently assessed for abnormalities of the tibial tuberosity because it is more associated with torsional deformity of the knees and trochlear dysplasia than the lateralization of the tibial tuberosity [15]. Second, the measurement of TT-TG requires the imaging data of computed tomographic (CT) and Magnetic Resonance Imaging (MRI). It seems better to select the most appropriate anatomical landmark for tibial rotational alignment by preoperative plans using preoperative CT or MRI directly [14]. Finally, it is impossible to measure TT-TG distance intraoperatively to evaluate the accuracy of tibial rotational axes in conventional total knee arthroplasty.

Seitlinger et al. [16] proposed a new method named as the tibial tuberosity–posterior cruciate ligament (TT-PCL) distance to assess the position of tibial tuberosity in which the medial border of the posterior cruciate ligament insertion and the midpoint of the patellar tendon at the attachment are viewed as anatomical landmarks. It was originally proposed as a risk factor for patellofemoral instability. The position of the tibial tuberosity has been shown to be more lateral in valgus knees than in varus knees [17]. However, it is not yet known whether the TT-PCL distance is associated with lateralization of the tibial tuberosity in varus knees. The tibial tuberosity and the posterior cruciate ligament insertion are not only both located on the same side of the knee, which is easy to be identified intraoperatively, but also consistent with the anatomic landmarks of tibial rotational axes. We hypothesized that the tibial tuberosity–posterior cruciate ligament (TT-PCL) distance can be used to evaluate the accuracy of the tibial rotational axes in total knee arthroplasty.

Therefore, the purpose of this study was to investigate: (1) whether TT-PCL distance in varus knees represents the true lateralization of tibial tuberosity in isolation; (2) how TT-PCL distance influences the accuracy of tibial rotational axes.

## Materials and methods

### Participants

One hundred and sixty-one osteoarthritis (Kellgren and Lawrence grade 3 or 4) patients with varus deformities who performed computed tomographic (CT) examination prior to primary TKA at our institution from February 2021 to March 2022 were investigated. The patients who met the following criteria were excluded from this study:(1) the patients whose anatomical landmarks cannot be accurately identified (*n* = 7) or with severe bone defects on the proximal tibia (*n* = 12); (2) hemophilic arthritis or rheumatoid arthritis (*n* = 4); (3) a history of knee trauma or infection (*n* = 3). At last, one hundred and thirty-five patients including 82 female and 53 male with varus deformities were enrolled in this study. The demographic data such as age, gender, hip-knee-ankle (HKA) angle and body mass index (BMI, kg/m2) of the 135 patients are listed in Table 1. The study was approved by local Medical Ethics Committee (XYFY2021-KL312-01).

**Table 1:** Demographic data of the patients

Parameter	Mean ± SD(range)
Age (years)	65.8 ± 7.7(43,89)
Gender (female/male)	82/53
BMI (kg/m2)	26.0 ± 3.4 (19.1,36.9)
KL grade (4/3)	102/33
Side (right/left)	71/64
HKA (°)	8.7 ± 5.3 (1.3,26.0)
Knee rotation (°)	3.16 ± 5.5 (-9.7, 14.4)
TT-PCL (mm)	19.2 ± 4.2 (10.5,28.6)
TW (mm)	74.0 ± 5.1 (61.9,86.7)
TTL distance (mm)	49.3 ± 6.1 (34.2,65.1)
TTL percentage (%)	66.5 ± 5.2 (54.7,82.4)
PCLL distance (mm)	44.0 ± 5.2 (29.7,55.3)
PCLL percentage (%)	59.4 ± 4.5 (47.5,70.4)
Mismatch angle of Akagi line and MTTT (°)	
Akagi line	-1.7 ± 5.3 (-22.4,9.2)
MTTT	7.6 ± 5.6 (-8.8,20.8)

### Radiology protocol and 3D model

The preoperative CT scans of the knees were performed with a GE LI-SPEED 16row CT scanner (General Electric Healthcare Corporation, Waukesha, WI, USA). The parameters were as the following: 512 × 512 matrix, 120 kV, 320 mA, 0.867 mm thickness, 0.867 mm skip between slices, and field of view of 14 cm × 14 cm. All lower limbs of the patients were in the neutral position and fully extended without internal or external rotation during CT scanning. Then the preoperative CT data was saved in DICOM (Digital Imaging and Communications in Medicine) format from the picture archiving and communication system (PACS) of our hospital. After that, all imaging data was imported into 3D reconstruction software (Mimics; Materialize, Leuven, Belgium) with initial processing. At last, a CAD software program (Solidworks; Dassault, Massachusetts, USA) was applied to reconstruct 3D knee models.

**Fig. 1:**
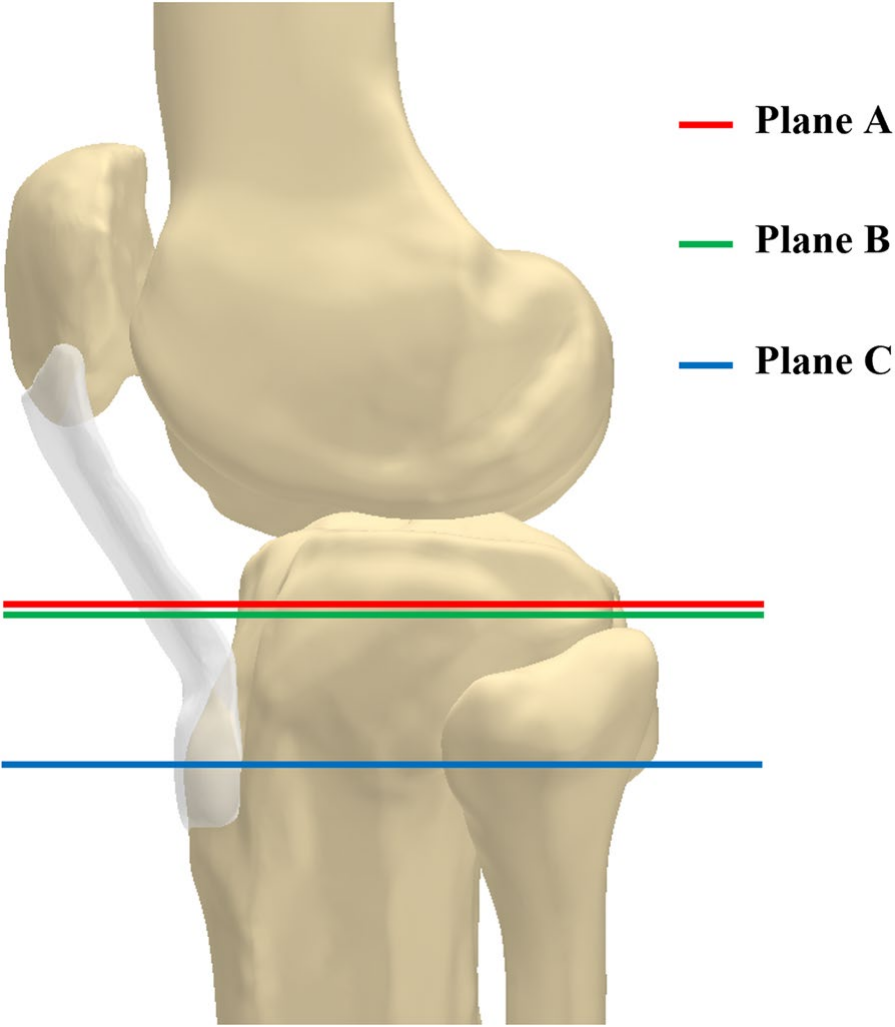
Three planes defined on the 3D models of the knee. Plane A (red): correlates with the tibial osteotomy plane, 8 mm distal to the center point of the lateral tibial plateau. Plane B (green): through the posterior cruciate ligament insertion at tibial condyle notch’s level. Plane C (blue): over the patellar tendon attachment’s level at tibial tuberosity

Three planes were defined on the 3D model of the knee to mark the anatomical landmarks correctly (Fig. 1). The plane A was defined as the tibial osteotomy plane which was taken as 8 mm distal to the center point of the lateral tibial plateau and perpendicular to the tibial anatomic axis. The simulation osteotomy of the tibial was performed with a posterior slope angle of 0° in the plane A. The plane B was defined as the level of the posterior tibial condyle notch where tibial posterior cruciate ligament inserted. The plane C was defined as the level where the patellar tendon attached the tibial tuberosity completely. All angles were defined as positive values if externally rotated with respect to the knee and negative values if internally rotated.

### Tibial rotational axes

**Fig. 2:**
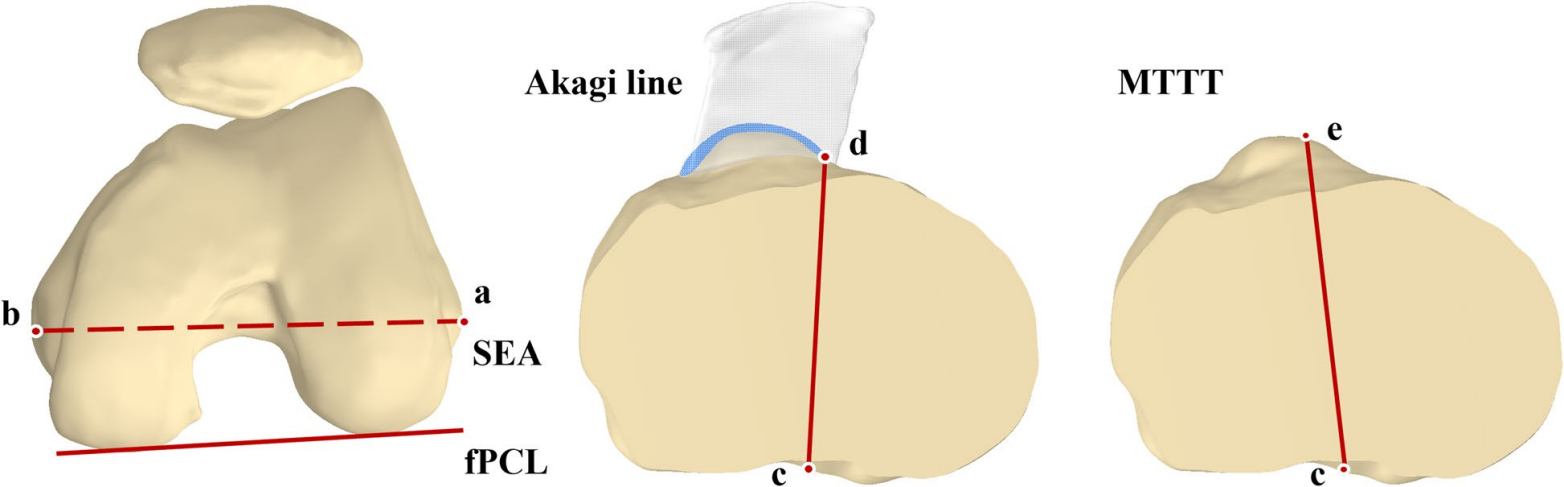
Rotational alignment axes for femoral and tibial component. The SEA axis (**a** and **b**): the line connecting the prominent point of lateral femoral epicondyle (**a**) and the medial femoral epicondylar sulcus (**b**). fPCL: the line tangent to the posterior border of the femoral posterior condyles. The Akagi line (**c** and **d**): the line connecting the midpoint of the posterior cruciate ligament insertion (**c**) and the medial border of the patellar tendon’s tibial attachment (**d**). MTTT (**c** and **e**): the line between the midpoint of the posterior cruciate ligament insertion (**c**) and the medial third of the tibial tuberosity (**e**)

Each anatomical landmark for rotational alignment was marked in the 3D model of the knee (Fig. 2). The prominent point of lateral femoral epicondyle and the medial femoral epicondylar sulcus were marked to establish the SEA which was projected into the plane A (Fig. 1). The line which was perpendicular to the projection of the SEA and passed through the middle point of the PCL insertion in the plane B was defined as anterior–posterior (AP) axis.

In the 3D model of the tibia, three anatomical landmarks were defined to establish three tibial rotational axes. The midpoint of the insertion of the posterior cruciate ligament, the medial border of the patellar tendon’s tibial attachment and the medial third of the tibial tuberosity were drawn and projected into the plane A. Then two tibial rotational axes were established: the Akagi line, the one between the midpoint of the posterior cruciate ligament insertion and the medial border of the patellar tendon’s attachment, and the line between the midpoint of the posterior cruciate ligament insertion and the medial third of the tibial tuberosity (MTTT) (Fig. 2).

The mismatch angles between the Akagi line and MTTT and the AP axis were measured by two experienced physicians respectively. The internal rotational angles of tibial rotational axes relative to the AP axis were recognized as negative values with the external rotational angles as positive values.

### Tibial tuberosity-posterior cruciate ligament distance

In this study, the TT-PCL distance was measured in the 3D model of the knee according to previous studies [18, 19]. The medial border of the posterior cruciate ligament insertion and the tibial posterior condyle line (tPCL) were drawn in the plane B. The medial edge of the tibial posterior condyle notch which was posterior to the medial intercondylar tubercle was viewed as the medial border of the posterior cruciate ligament insertion [18]. The line tangent to the posterior border of the medial and lateral tibial condyles was viewed as the posterior tibial condyle line. Two lines which were perpendicular to tPCL and passing through the projection of the medial border of the posterior cruciate ligament insertion and the projection of the midpoint the patellar tendon’s attachment, respectively, were drawn in plane B. The distance between the two lines was defined as tibial tuberosity -posterior cruciate ligament distance (Fig. 3.1).

**Fig. 3:**
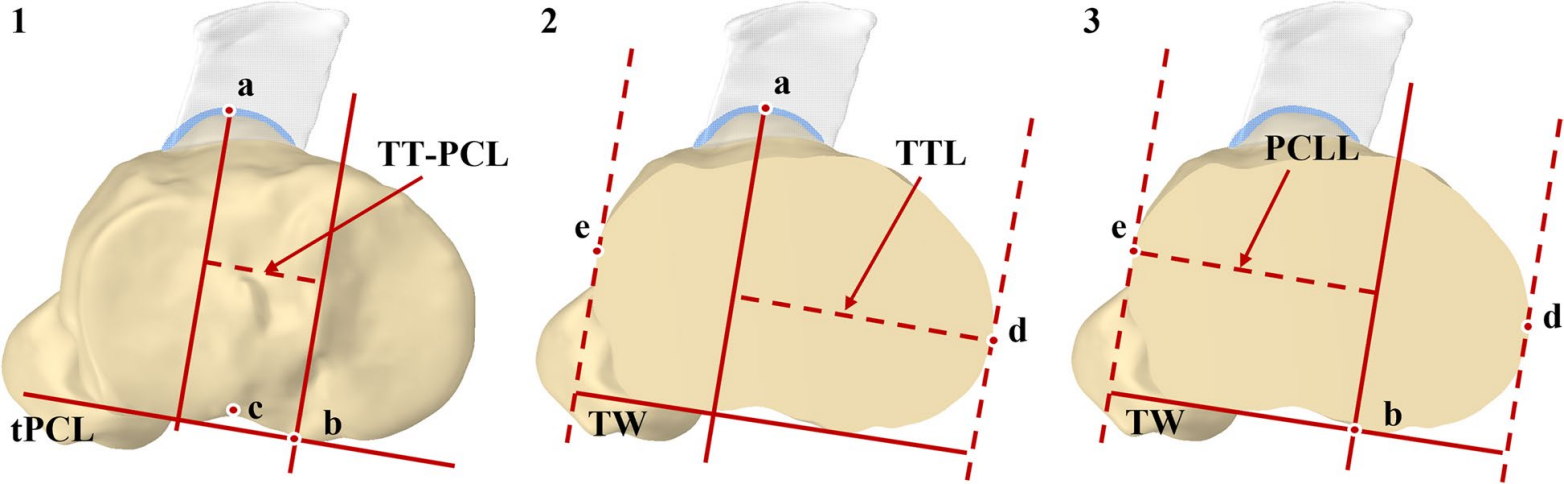
Tibial anatomical parameters. Figure 3.1 TT-PCL distance: the distance between the lines which were simultaneously perpendicular to tPCL and passing through the projection of the medial border of the posterior cruciate ligament insertion (**b**) and the projection of the midpoint of the patellar tendon’s attachment (**a**) respectively. The medial and lateral borders of the posterior cruciate ligament insertion (**b** and **c**). Figure 3.2 TW: the distance between the lines that were perpendicular to the tPCL and tangent to the tibial medial–lateral borders (**d** and **e**); TTL distance: the distance between the projection of the midpoint of the patellar tendon’s attachment (**a**) and the medial border of the proximal tibia (**d**). Fig. 3.3 PCLL distance: the distance between the projection of the medial border of the posterior tibial condyle notch (**b**) and the lateral border of the proximal tibia (**e**)

### Tibial tuberosity lateralization and posterior cruciate ligament lateralization

In this study, the position of the tibial tuberosity relative to the medial–lateral dimension of proximal tibia was assessed using the tibial tuberosity lateralization according to the method of Tensho et al. [15]. The width of the proximal tibia (TW distance) was defined as the distance between the lines that were perpendicular to the tPCL and tangent to the tibial medial–lateral borders in plane B. The tibial tuberosity lateralization distance (TTL distance) was defined as the distance between the projection of the midpoint of the patellar tendon’s attachment and the medial border of the proximal tibia in plane A (Fig. 3.2). The posterior cruciate ligament lateralization distance (PCLL distance) was the distance between the projection of the medial border of the posterior tibial condyle notch and the lateral border of the proximal tibia in plane B (Fig. 3.3). The tibial tuberosity lateralization (TTL) was defined as the TTL distance/TW distance, and the posterior cruciate ligament lateralization (PCLL) was defined as PCLL distance / TW distance.

### Knee rotation angle

**Fig. 4:**
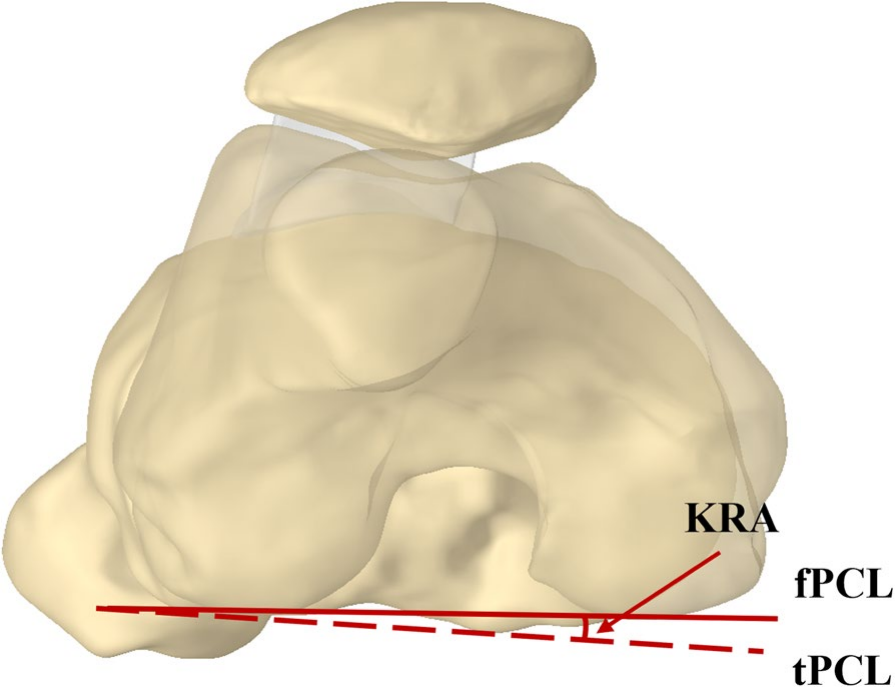
knee rotational angle. The angle between the fPCL and the tPCL was defined as knee rotation angle (KRA)

The relative relationship between the distal femur and the proximal tibia represented the knee rotation which was assessed by the torsional angle of the femur and tibial posterior condyle. A line tangent to the posterior border of the femoral posterior condyles which was defined as the femoral posterior condyle line (fPCL) was made in the femoral 3D models and projected in the plane B. The angle between the fPCL and the tPCL was the knee rotation angle (KRA) (Fig. 4).

### Statistical analysis

All statistical analyses were processed by SPSS statistical software (version 26.0; SPSS Inc, Chicago, IL, USA). The normality of all data measured by two independent researchers was tested by the Kolmogorov–Smirnov normality test. The measurements were performed again one month after the initial measurements by one of the physicians to assess the intraclass correlation coefficients (ICC). The sample of the study was calculated to be at least 132 cases using G*Power 3.1 (effect size 0.3, power 0.95, and alpha 0.05).

At first, the correlations between TT-PCL with tibial anatomical parameters and the mismatch angles of the Akagi line and MTTT were analyzed using the Pearson correlation coefficient. The r-value of the correlation coefficient was classified as the following: strong (≥ 0.7), moderate (0.4–0.7), or mild (0.2–0.4). Then, the t-test was used to compare the mismatch angle between the Akagi line and MTTT and the AP axis, respectively. The distributions of the mismatch angles of the Akagi line and MTTT with the safe zone (-5°-10°) were counted. Thirdly, ROC-curve analysis was used to identify the cut-off points of the TT-PCL distance for the Akagi line and MTTT according to the distributions of the mismatch angles with the safe zone (-5°-10°), respectively. The cut-off values of TT-PCL for the Akagi line and MTTT were calculated by the Youden’s index. An optimal cut-off value of TT-PCL was determined in the combined use of the Akagi line and MTTT for tibial rotational alignment. A *p* value < 0.05 was considered to be statistically significant.

## Results

All data from both physicians was tested for consistency prior to the analysis. Both Intra- and interobserver ICC values were greater than 0.8, indicating high confidence in the measurements. Table 1 shows the results of all measurements of a total of 135 patients. The Kolmogorov–Smirnov normality test showed that all data conformed to a normal distribution.

The correlations between TT-PCL distance and tibial anatomical parameters and tibial rotational axes are shown in Table 2. TT-PCL distance was significantly and moderately correlated with TTL (*r* = 0.547, *p* < 0.001) and TW(*r* = 0.484, *p* < 0.001). TT-PCL distance was significantly and mildly correlated with PCLL (*r* = 0.344, *p* = 0.096). There was no significant association between TT-PCL distance and KRA. The Akagi line and MTTT were in the moderately strong and significant correlation with TT-PCL distance(*r* = 0.450, *p* < 0.001; *r* = 0.577, *p* < 0.001).

**Table 2:** The Pearson’s correlation between TT–PCL and tibial anatomical parameters and tibial rotational axes

		Tibial anatomical parameters				Tibial rotational axes	
		TW	TTL	PCLL	KRA	Akagi line	MTTT
TT-PCL	r	0.484	0.547	0.344	0.001	0.450	0.577
	*p*	< 0.001	< 0.001	0.096	0.991	< 0.001	< 0.001

**Fig. 5:**
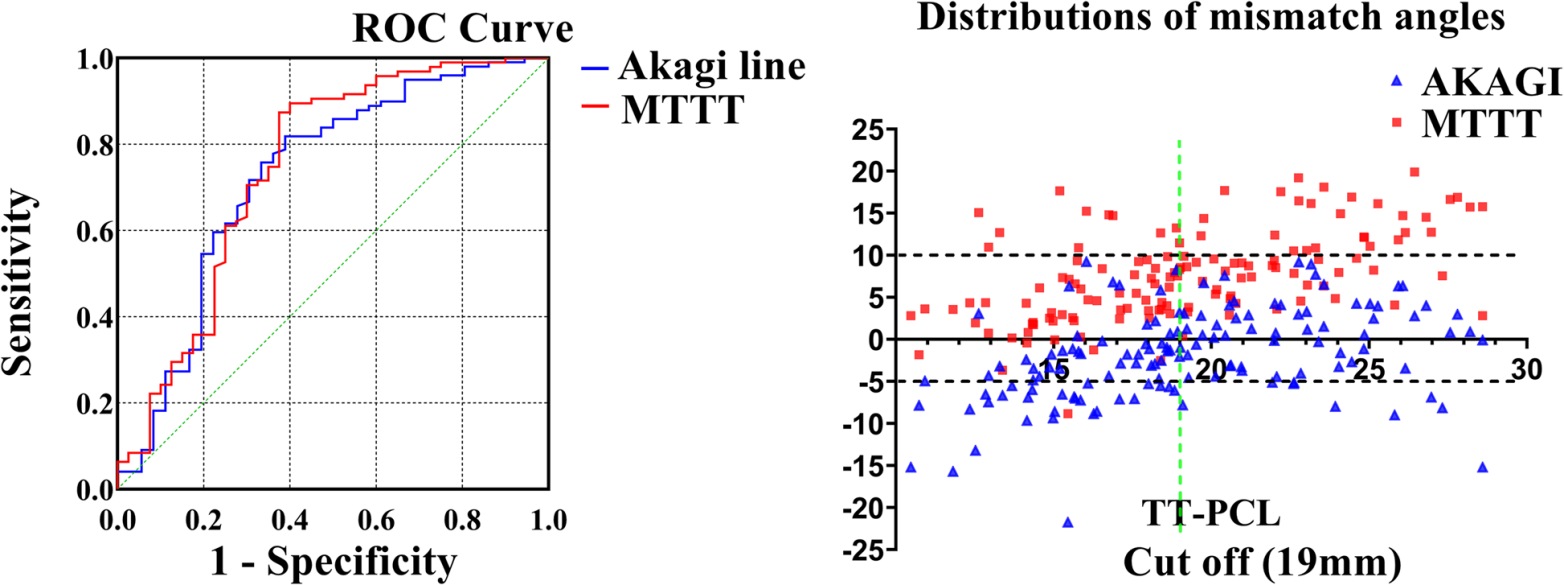
**A** ROC curve of the Akagi line and MTTT; **B** the frequency distributions of the mismatch angles with the cut-off value 19 mm of TT-PCL

The Akagi line was internally rotated by -1.7° ± 5.5° relative to the AP axis, while the MTTT was externally rotated by 7.6° ± 5.6° relative to the AP axis. Both were significantly different from the AP axis (*P* < 0.001). The cases which were inside the safe zone were 99 cases (73.3%) and 95 cases (70.4%) in the patients when the Akagi line and MTTT were referenced as tibial rotational axes. Based on the analysis of ROC curve, the cut-off point of TT-PCL distance was 16.46 with an area under curve (AUC) of 0.73 (95% CI 0.62–0.83) for the Akagi line and 22 with an AUC of 0.75 (95% CI 0.64–0.85) for MTTT (Fig. 5).

The distributions of the mismatch angles with the cut-off values of TT-PCL distance from 16 to 22 mm were calculated when used the Akagi line and MTTT for tibial rotational alignment in combination (Table 3). Regardless of the application of the cut-off value that ranged from 16 to 22 mm, the combination of tibial rotational axes was less likely to cause the tibial component outside the safe zone compared to the single one referenced. The percentage of the cases inside the safe zone increased from the lowest to the highest as the cut-off value of TT-PCL distance increased from 16 to 19 mm. When the cut-off value of TT-PCL distance was 19 mm, a total of 115 cases (85.2%) were inside the safe zone (Fig. 5). Afterwards, as the cut-off value of TT-PCL distance further increased to 22 mm, the percentage of the cases inside the safe zone decreased. When the TT-PCL distance was greater than 19 mm, the mismatch angles of Akagi line and MTTT axis were 0.5° ± 4.4°and 10.3° ± 4.6°, respectively. The percentage of the cases referring to the Akagi line inside the safe zone was 87.3%. When the TT-PCL distance was less than 19 mm, the mismatch angles of Akagi line and MTTT axis were -3.6° ± 5.4°and 5.3° ± 4.9°, respectively. The percentage of the cases referring to MTTT inside the safe zone was 83.3%.

**Table 3:** The distribution of the mismatch angles of tibial rotation axes with different cut-off values of TT-PCL distance

Cut-off value(mm)		Akagi line		MTTT		combination	
		Inside safe zone	Outside safe zone	Inside safe zone	Outside safe zone	Inside safe zone	Outside safe zone
16	Lower(*n* = 36)	17 (47.2%)	19 (51.8%)	30 (83.3%)	6 (16.7%)	112 (83.0%)	23 (17.0%)
	Upper(*n* = 99)	82 (82.8%)	17 (17.2%)	65 (65.7%)	34 (34.3%)		
17	Lower(*n* = 43)	21(48.8%)	22 (51.2%)	34 (79.1%)	9 (20.9%)	112 (83.0%)	23 (17.0%)
	Upper(*n* = 92)	78 (84.8%)	14 (15.2%)	61 (66.3%)	31 (33.7%)		
18	Lower(*n* = 52)	28 (53.8%)	24 (46.2%)	43 (82.7%)	9 (17.3%)	114 (84.4%)	21 (15.6%)
	Upper(*n* = 83)	71 (85.5%)	12 (14.5%)	52 (62.2%)	31 (37.8%)		
19	Lower(*n* = 72)	44 (61.1%)	28 (38.9%)	60 (83.3%)	12 (16.7%)	115 (85.2%)	20 (14.8%)
	Upper(*n* = 63)	55 (87.3%)	8 (12.7%)	33 (55.6%)	28 (44.4%)		
20	Lower(*n* = 82)	53 (64.6%)	29 (35.4%)	68 (82.9%)	14 (17.1%)	114 (84.4%)	21 (15.6%)
	Upper(*n* = 53)	46 (86.8%)	7 (13.2%)	27 (50.9%)	26 (49.1%)		
21	Lower(*n* = 94)	65 (69.1%)	29 (30.9%)	79 (84.0%)	15 (16%)	113 (83.7%)	22 (16.3%)
	Upper(*n* = 41)	34 (82.9%)	7 (17.1%)	16 (39.0%)	25 (61.0%)		
22	Lower(*n* = 98)	68 (69.4%)	30 (30.6%)	83 (84.7%)	15 (15.3%)	114 (84.4%)	21 (15.6%)
	Upper(*n* = 37)	31 (83.8%)	6 (16.2%)	12 (32.4%)	25 (67.6%)		

## Discussion

The meaningful findings demonstrated in this study were as follows: 1) TT-PCL distances cannot reflect the true lateralization of tibial tuberosity in isolation.2) As the TT-PCL distance increases, the rotational alignment axes for tibial component will tend to be externally rotated.3) It is recommended to apply 19mm as the cut-off value for TT-PCL distance to adjust the choice of tibial rotational axes. The Akagi line can be referred when TT-PCL distance>19 mm and MTTT can be referred when TT-PCL distance≤19 mm.

Despite the advances of the surgical techniques and the designs for components in total knee arthroplasty, there are still approximately 20% of the osteoarthritis patients dissatisfied with their clinical outcomes [20]. One of the main causes of this condition is the unexplained pain following total knee arthroplasty [21]. It has been demonstrated that excessive internal rotation of the tibial components is an important risk factor for postoperative pain [22, 23]. After comparing patients in the painful and asymptomatic groups after TKA, Bell [24] et al. concluded that excessive internal rotation of the tibial component up to 5.8° was an important cause of unexplained pain following TKA, whereas external rotation of the tibial component did not seem to be associated with the postoperative persistent pain. However, it does not mean that we advocate unrestricted external rotation placement of the tibial component because rotational alignment of tibial component is not an isolated factor and affects tibial coverage on the cutting plane. Due to the asymmetric anatomical morphology of the tibial plateau, if the surgeon focuses on the effect of rotational alignment of the tibial component on the patients' postoperative function while ignoring the coverage of the tibial osteotomy surface, it is likely to result in posterolateral overhang of tibial component on the osteotomy surface which can also negatively affect the clinical outcome after TKA [25, 26]. This implies that a compromise between rotational alignment of the tibial component, tibial coverage, and the incidence of posterolateral overhang need to be reached. Simsek et al [26] referenced the medial third of the tibial tuberosity to measure the malrotation of tibial components and counted the incidence of tibial overhang in the patients postoperatively. It was proved that external rotation of the tibial components beyond 2.6° relative to MTTT increased the incidence of posterolateral overhang. Therefore, a range of mismatch angle from -5° to 10° was considered as a safe zone for rotational alignment of the tibial component.

In order to install tibial components in the appropriate position on the horizontal plane, many anatomical landmarks have been proposed to construct tibial rotational axes. Insall et al. [27] first found that maximum functionalization of the knee could be achieved with reference to the medial third of the tibial tuberosity in the absence of abnormal changes in the tibial tuberosity. However, this technique is not supported by a relevant theoretical background, relies mainly on the clinical experience of the operator, and is not very reliable [13, 14, 28]. Akagi et al. [7] found that the line from the midpoint of the posterior cruciate ligament insertion to the medial border of the patellar tendon at the attachment were essentially parallel to the AP axis with an average angle of 0.0° ± 2.8° in healthy volunteers. However, its performance in osteoarthritis patients is not as good as in the healthy [9, 14]. Lu et al. [28]found that the angles of MTTT and the Akagi line relative to the line perpendicular to the SEA were 11.9° ± 5.4° and 1.4° ± 5.0°, respectively. In this study, MTTT was also found to be externally rotated than the Akagi line. The one difference was that the mismatch angle between rotational axes and the SEA in this study was more internal when compared to the previous study [28]. The disagreement may be contributed to two factors. On the one hand, the difference of measurements between 3D reconstruction and 2D-CT may be the cause [29]. On the other hand, the flexion constraints leading to tibial internal rotation was neglected in this study which might have influence on the accuracy of the measurements [9]. In this study, MTTT externally rotated by 7.6° ± 5.6° relative to the AP axis. There were 95 cases (70.4%) inside the safe zone when MTTT was referenced as tibial rotational axes. Based on the analysis of ROC curve, the cut-off point of TT-PCL distance was 22mm for MTTT. When TT-PCL distance<22mm,84.7% of the135 cases were inside the safe zone. It was proved that even though the tibial component installed with reference to MTTT may result in excessive external rotation, the probability of the tibial component in the proper position increased when the position of tibial tuberosity was considered. The Akagi line internally rotated by -1.7° ± 5.5° relative to the AP axis as previous study [9, 28]. The cases inside the safe zone were 99 cases (73.3%). The cases of tibial components outside the safe zone were not significantly less when referring to the Akagi line than when referring to the MTTT despite the higher accuracy of the Akagi line. Based on the analysis of ROC curve, the cut-off point of TT-PCL distance was 16.46mm for the Akagi line. When TT-PCL distance>16.46mm,the percentage of the cases inside the safe zone increased to 85.3% .

One important finding of this study was that there were positive relationships between TT-PCL distance and the mismatch angle of tibial rotational axes relative to the SEA, though TT-PCL distance in varus knees is affected not only by the lateralization of tibial tuberosity, but also by the lateralization of posterior cruciate ligament insertion and proximal tibial width. In this study, the mean TT-PCL distance was 19.2 ± 4.3 mm, which was smaller than the previous measurement based on 3D models [19]. The difference may be caused by the factor of ethnicity [30]. An optimal cut-off value (19 mm) of TT-PCL distance was found in the safe zone (-5°-10°) to combine the selection of the Akagi line and MTTT. It is advisable to reference the medial border of the patellar tendon at the attachment when TT-PCL > 19 mm and the medial third of the tibial tuberosity when TT-PCL ≤ 19 mm.The combination use of anatomical landmarks may reduce the incidence of tibial components outside the safe zone.

This study has the following limitations: First, this study investigated the effect of the position of tibial tuberosity on the accuracy of tibial rotational alignment in Eastern population based on 3D reconstruction technology. The findings of the study may not be applicable to western populations. Second, this study enrolled the OA patients with varus knees excluding those with valgus knees. The influences of the valgus deformity of the knees on tibial rotation alignment and TT-PCL distance were not taken into consideration. At the same time, the flexion constraints leading to tibial internal rotation was neglected in this study which might have influence on the accuracy of the measurements. Third, this study derived the cut-off values of TT-PCL distance to reference different anatomical landmarks based on a safe zone (-5°-10°) generalized from previous studies [24–26] but did not actually calculate the incidence of posterolateral overhang of the tibial components when the Akagi line and MTTT were applied as tibial rotational axes, nor did it consider the effect of symmetrical and asymmetrical design of tibial components on the results. Lastly, since the enrolled were the OA patients with Kellgren-Lawrence grade 3 or 4, many patients had osteophytes on the femur and tibia. Though the osteophytes have been dealt with in 3D software, it may still have influence on the accuracy of the measurements on anatomical parameters. Nevertheless, this study proposed the cut-off value of TT-PCL distance based on 3D reconstruction technique to combine the application of the Akagi line and MTTT to help orthopaedical surgeons select appropriate landmarks of tibial tuberosity intraoperatively to improve the accuracy of rotational alignment of tibial components.

## Conclusions

TT-PCL distances cannot reflect the true lateralization of tibial tuberosity in isolation but can aid in the combination of the Akagi line and MTTT in varus knees. The patients with TT-PCL distance > 19 mm are recommended to reference the Akagi line for tibial rotational alignment. MTTT is recommended to the patients with TT-PCL distance ≤ 19 mm. The study will aid surgeons in deciding which reference may be used by measuring TT-PCL distance using a preoperative CT.

## Data Availability

The datasets generated and/or analyzed during the current study are not publicly available due institutional privacy guideline but are available from the corresponding author on reasonable request.

## Abbreviations

TT-PCL distance: Tibial tuberosity–posterior cruciate ligament distance
MTTT: Medial third of the tibial tuberosity
TW: Tibial width
TTL: Tibial tuberosity lateralization
PCLL: Posterior cruciate ligament lateralization
KRA: Knee rotation angle
ROC curve: Receiver operating characteristic curve
SEA: Surgical epicondylar axis
AUC: Area under curve
3D: Three-dimension
